# County- and State-level Estimates of Population Sizes of Men Who Have Sex With Men Across the United States

**DOI:** 10.1093/ofid/ofag148

**Published:** 2026-04-14

**Authors:** Duygu Islek, Stephanie DuBose, Gordon Le, Eric W Hall, Patrick S Sullivan

**Affiliations:** Department of Epidemiology, Rollins School of Public Health, Emory University, Atlanta, Georgia, USA; Department of Epidemiology, Rollins School of Public Health, Emory University, Atlanta, Georgia, USA; Department of Epidemiology, Rollins School of Public Health, Emory University, Atlanta, Georgia, USA; OHSU-PSU School of Public Health, Oregon Health & Science University, Portland, Oregon, USA; Department of Epidemiology, Rollins School of Public Health, Emory University, Atlanta, Georgia, USA

**Keywords:** decennial census, geographic distribution, men who have sex with men (MSM), NHANES, population estimates

## Abstract

**Background:**

Accurate estimates of the population sizes of men who have sex with men (MSM) are essential for evaluating HIV-related interventions. We aimed to estimate MSM populations at county and state levels using recent data from the decennial census and the National Health and Nutrition Examination Survey.

**Methods:**

We used 2020 Decennial Census data to calculate a weight for each U.S. county, reflecting the proportion of male-male partner households relative to counties of similar urbanicity based on the 2013 National Center for Health Statistics Urban–Rural Classification Scheme. We applied these weights to urbanicity-stratified estimates of the prevalence of adult men who reported sex with a man in the past 12 months, derived from National Health and Nutrition Examination Survey (2015–2020). Multiplying these percentages by adult male populations produced county estimates, which were aggregated to state levels.

**Results:**

We estimated approximately 2.1 million MSM in the United States, representing 1.7% of adult males. State-level estimates ranged from 0.3% in Wyoming (n = 675) to 3.7% in the District of Columbia (n = 9709). California had the largest MSM population (n = 318 612; 2.1%), followed by Florida (n = 191 199; 2.3%) and Texas (n = 173 751; 1.6%). At the county level, Los Angeles County, CA, had the largest MSM population (n = 82 513; 2.1%), followed by Cook County, IL (n = 39 580; 2.0%), and Broward County, FL (n = 34 321; 4.7%). Broward County, FL (4.7%), and San Francisco County, CA (4.6%), had the highest proportions relative to their male populations.

**Conclusions:**

County- and state-level MSM estimates provide crucial denominators for calculating disease rates and informing public health interventions.

In the United States, the transmission of HIV through male-male sexual contact is highly prevalent [1]. To assess the success of HIV-related health interventions, it is crucial to have estimates of the population sizes of men who have sex with men (MSM) at both national and local levels [2, 3]. Although HIV surveillance data provide estimates of the number of new cases (serving as the numerator), determining disease rates for national, regional, and finer geographic levels requires estimates of MSM population sizes to calculate the denominators (number of MSM by geographic levels of interest) for disease rates [4, 5].

To assess HIV prevalence and incidence at more localized levels and to compare the HIV burden among MSM in various regions, it is necessary to use estimation methods at finer scales [5]. However, large-scale surveys like the U.S. decennial census do not typically collect detailed demographic data on sexual behavior, particularly same-sex behavior, at the state or county level. This lack of data makes it challenging to estimate the prevalence or incidence of HIV and other sexually transmitted infections (STIs) among MSM at these levels. Consequently, indirect methods for estimating the population sizes of MSM are crucial to provide denominators for calculating rates across different geographic areas, particularly for effective state and local HIV prevention planning [6].

Our research team previously developed a novel method for estimating the population sizes of MSM across US states, counties, and core-based statistical areas (CBSAs) [7–10]. However, to remain useful, these estimates need to be regularly updated through incorporating the latest data estimates. This is necessary to provide public health professionals and policymakers with timely denominators for assessing recent disease rates at both state and county levels, including in nonurban regions. Therefore, in the current study, we aimed to estimate the sizes of MSM populations at the county level indirectly, incorporating the latest data estimates from 2020 Decennial Census [11] and National Health and Nutrition Examination Survey (NHANES) 2015–2020 March [12] and to aggregate these county-level estimates to form state and CBSA totals.

## METHODS

### Data Sources

This analysis will use methodology previously published by members of our research team [7]. This analysis serves as an update to the previous work by incorporating the most recent data from 2020 Decennial Census [11] and NHANES 2015–2020 March cycles [12]. The full methodology is previously described elsewhere [7].

We used data from the 2020 Decennial Census demographic and housing statistics, to obtain the total number of households, total number of male-male partner households and total number of men aged 18 years and older for each county in the United States [11] (Supplementary Table 1). The 2020 Census Demographic and Housing Characteristics data file is publicly available [11]. The data files provide deidentified and unaggregated data on total number of households, total number of male-male married and unmarried partner households for each county in the United States. We did not include data from US territories. The methodology of 2020 Decennial Census is described elsewhere [13].

To estimate the weighted percentages (prevalence) of MSM and confidence intervals overall and separately stratified by urbanicity and by US census region, we combined publicly available demographic data with urbanicity-level sexual behavior data from the restricted NHANES (2015–March 2020) data files [12] to describe the prevalence of men who had oral or anal sex with at least 1 man in the past 12 months and to describe the prevalence of men who had ever had oral or anal sex with a man, respectively (Supplementary Table 2, Supplementary Table 3).

To determine the urbanicity level of a county, we used the 2013 Centers for Disease Control and Prevention National Center for Health Statistics Urban-Rural Classification Scheme for Counties [14]. In this classification scheme, counties fall into 6 categories: central (ie, inner city) or fringe (ie, suburban) portions of large metropolitan statistical areas (population size ≥ 1 000 000), medium-sized metropolitan statistical areas (population size of 250 000–999 999), small metropolitan statistical areas (population of < 250 000), micropolitan area (counties that contain all or part of a city of 10 000 or more), and noncore (counties that do not contain any part of a city of 10 000 or more) [4]. To have the required minimum cell sizes to avoid any confidentiality disclosure risk, we collapsed “small metro, metropolitan, and noncore” into 1 urbanicity level.

Data analysis was conducted at the county level and then compiled to reflect state level and the Office of Management and Budget's CBSAs, which encompass both metropolitan and micropolitan statistical regions. Metropolitan statistical areas are CBSAs with populations of at least 50,000, whereas micropolitan statistical areas have populations below 50 000.

### Statistical Analysis

The analysis is summarized in 4 steps.

**Figure 1.**
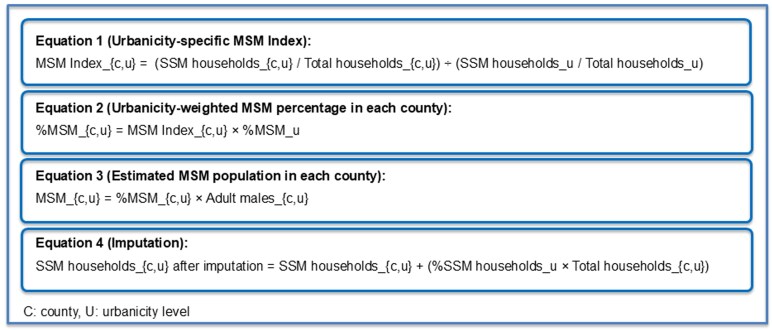
Equations used for calculations.

In the first step, for each county in the United States, we estimated an MSM Index that represents the relative representation of same-sex male (SSM) households compared to the national average within a respective level of urbanicity (the degree to which a geographic area is urban) (Equation 1, Figure 1). For this step, we used the 2020 Decennial Census to obtain total number of households, total number of SSM households and total number of men aged 18 years and older for each county in the United States [11]. We first divided the number of SSM households with the total number of households in a county. We then divided this result with the urbanicity-stratified national proportion of SSM households among all households in the United States, to arrive at the MSM index (Equation 1, Figure 1).

As the second step in our analysis, we used the NHANES data (2015–2020 March cycles) [12] to calculate weighted percentages and confidence intervals of MSM overall and stratified by urbanicity and by US census region as designated by the US Census Bureau: Northeast, Midwest, South, and West [15]. NHANES does not have data on every county in the United States and is not designed for representative county-level direct estimates. We examined prevalence of the following separately by urbanicity and region: (1) having had oral or anal sex with another man during the past 12 months or (2) having ever had oral or anal sex with another man. All estimates were weighted to account for selection probabilities, nonresponse, and coverage. Only the urbanicity-stratified estimates were used in the county-level modeling. Because this analysis used geographic and sexual behavior variables that are not accessible in the public dataset, analysis was carried out at a federal Research Data Center in Atlanta, GA [16].

As the third step, we multiplied the within-stratum MSM index (from step 1) by the estimated percentage of MSM by urbanicity level (from step 2). This methodology applied urbanicity-stratified prevalence estimates, consistent with the construction of the MSM index. Regional stratification was examined descriptively but not incorporated into the weighting equations. We then arrived at the percentage of males who are MSM in each county/county cluster (Equation 2, Figure 1). As the final step, we multiplied the MSM percentage by the total adult males to estimate the number of MSM in each county/county cluster (Equation 3, Figure 1).

To account for counties with no reported SSM households, we used an imputation method that added a small number of SSM households based on urbanicity-specific averages. Specifically, we added SSM households to both the numerator and denominator of the index equation based on urbanicity-specific SSM percentages [7] (see Figure 1, Equation 4). This adjustment preserved relative differences across counties while ensuring that areas with no reported SSM data still contributed to MSM estimates. Because the MSM Index was used for weighing rather than direct estimation, this imputation did not inflate final population estimates. We found that MSM population estimates showed instability and outliers in counties with fewer than 550 adult males; thus, we excluded these counties from the analysis. Finally, we aggregated county-level data to have state- and CBSA-level MSM population estimates.

Although this analysis builds on a previously published method developed by our group, several methodological elements differ from our team's earlier work (Grey et al. 2016). First, our prior study applied behavioral prevalence estimates based on a 5-year recall period for male–male sexual behavior, derived from American Community Survey. However, the present analysis uses restricted-use NHANES sexual behavior data (2015–March 2020) to derive prevalence estimates based on 2 alternative definitions: (1) sex with a man in the past 12 months and (2) ever having had sex with a man. This is the first time our group has used restricted NHANES sexual behavior data as the behavioral input for this estimation framework. In addition, because of confidentiality constraints and minimum cell size requirements within restricted NHANES data, we collapsed small metropolitan, micropolitan, and noncore counties into a single urbanicity category, whereas earlier analyses retained finer stratification. All other components of the analysis including construction of the MSM index using census same-sex male household data and application of urbanicity-weighted prevalence estimates remain conceptually consistent with prior work.

Data were analyzed using statistical software package SAS version 9.4 (SAS Institute, Cary, North Carolina).

## RESULTS

Prevalence estimates of MSM in the past 12 months and men who had ever had sex with men using NHANES 2015–2020 March [12] are presented in Table 1. Using prevalence estimates of MSM in the past 12 months, we found that 2 115 486 MSM reside in the United States, comprising 1.7% of the adult male population nationwide (**Table 2**). California had the highest number of MSM (n = 318 612), accounting for 15.1% of the national MSM population. This was followed by Florida (n = 191 199; 9.0%) and Texas (n = 173 751; 8.2%). The District of Columbia had the highest proportion of MSM relative to its adult male population at 3.7%. States with the lowest estimated MSM counts included South Dakota (n = 988), North Dakota (n = 892), and Wyoming (n = 675).

**Table 1.** Prevalence of Male-male Sex, by Urbanicity and US Census Region, National Health and Nutrition Examination Survey, 2015–March 2020

		Ever Had Male-male Sex	Had Male-male Sex In Past 12 mo
	n	% (95% CI)	% (95% CI)
**Overall**	7116	8.72 (6.83–10.61)	1.75 (1.03–2.47)
**Urbanicity**			
Large central metro	2290	11.13 (6.73–15.53)	2.04 (0.82–3.26)
Large fringe metro	1359	8.91 (5.21–12.61))	2.16 (0.46–3.87)
Medium metro	1969	7.88 (3.28–12.48)	2.14 (0.0–4.49)
Small metro, micropolitan and noncore (collapsed)	1498	5.71(2.97–8.46)	0.33 (0.00–0.85)
**Region**			
Northeast	1064	6.77 (1.99–11.54)	1.8 (0.0–4.05)
Midwest	1383	10.26 (3.84–16.69)	0.94 (0.18–1.70)
South	3328	9.42 (6.50–12.34)	2.39 (1.34–3.43)
West	1341	7.73(4.10–11.37)	1.15 (0.00–2.70)

Abbreviation: CI, confidence interval.

**Table 2.** Estimated MSM Populations in 50 States and the District of Columbia, Ranked by Size of MSM Population Using Prevalence Estimates of Men Who had Sex With Men in Past 12 Months^a^

Rank	State	Adult Males (n)	MSM (n, %)	% of Total MSM
1	California	15 092 993	318 612 (2.1)	15.1
2	Florida	8 318 714	191 199 (2.3)	9.0
3	Texas	10 676 141	173 751 (1.6)	8.2
4	New York	7 666 027	148 875 (1.9)	7.0
5	Pennsylvania	5 005 473	80 621 (1.6)	3.8
6	Illinois	4 845 934	76 866 (1.6)	3.6
7	New Jersey	3 492 639	68 895 (2.0)	3.3
8	Georgia	3 918 344	66 194 (1.7)	3.1
9	Ohio	4 456 327	64 074 (1.4)	3.0
10	Massachusetts	2 702 673	62 898 (2.3)	3.0
11	North Carolina	3 901 719	58 433 (1.5)	2.8
12	Washington	2 983 509	57 020 (1.9)	2.7
13	Virginia	3 255 740	53 288 (1.6)	2.5
14	Michigan	3 864 050	51 410 (1.3)	2.4
15	Maryland	2 279 740	46 848 (2.1)	2.2
16	Arizona	2 715 833	46 006 (1.7)	2.2
17	Colorado	2 250 459	44 118 (2.0)	2.1
18	Tennessee	2 587 094	36 201 (1.4)	1.7
19	Indiana	2 529 026	32 279 (1.3)	1.5
20	Missouri	2 317 242	30 698 (1.3)	1.5
21	South Carolina	1 910 038	30 387 (1.6)	1.4
22	Minnesota	2 161 621	28 796 (1.3)	1.4
23	Oregon	1 652 902	28 256 (1.7)	1.3
24	Connecticut	1 373 574	27 481 (2.0)	1.3
25	Wisconsin	2 275 140	24 476 (1.1)	1.2
26	Louisiana	1 707 087	23 910 (1.4)	1.1
27	Nevada	1 198 598	23 147 (1.9)	1.1
28	Oklahoma	1 475 525	19 498 (1.3)	0.9
29	Kentucky	1 691 374	19 096 (1.1)	0.9
30	Alabama	1 861 340	18 997 (1.0)	0.9
31	Utah	1 156 059	17 941 (1.6)	0.8
32	Kansas	1 099 110	13 202 (1.2)	0.6
33	Arkansas	1 118 227	13 167 (1.2)	0.6
34	Hawaii	573 981	12 374 (2.2)	0.6
35	Iowa	1 207 151	11 910 (1.0)	0.6
36	New Mexico	799 990	11 344 (1.4)	0.5
37	District of Columbia	264 627	9709 (3.7)	0.5
38	Mississippi	1 081 825	9609 (0.9)	0.5
39	Nebraska	723 943	9592 (1.3)	0.5
40	Delaware	371 822	9176 (2.5)	0.4
41	New Hampshire	550 142	8721 (1.6)	0.4
42	Rhode Island	424 360	8604 (2.0)	0.4
43	Maine	538 251	7401 (1.4)	0.3
44	Idaho	681 530	6666 (1.0)	0.3
45	West Virginia	703 779	5005 (0.7)	0.2
46	Alaska	283 116	3688 (1.3)	0.2
47	Montana	421 606	1275 (0.3)	0.1
48	Vermont	256 089	1217 (0.5)	0.1
49	South Dakota	332 921	988 (0.3)	0.0
50	North Dakota	303 212	892 (0.3)	0.0
51	Wyoming	222 567	675 (0.3)	0.0
	Total	125 281 184	2 115 486 (1.7)	

Abbreviations: MSM, men who have sex with men; NHANES, National Health and Nutrition Examination Survey.

^a^State-level estimates were created by applying NHANES urbanicity-level estimates of men who have sex with men to 2020 Decennial Census county-level estimates of same-sex male households.

At the county level, we ranked 50 counties by estimated MSM population using prevalence estimates of men who had sex with men in the past 12 months (Table 3). Los Angeles County, CA, had the largest absolute number of MSM at 82 513 (2.1% of adult males), followed by Cook County, IL (n = 39 580; 2.0%), and Broward County, FL (n = 34 321; 4.7%). Notably, Broward County, FL, and San Francisco County, CA, had among the highest proportions of MSM relative to adult males as 4.7% and 4.6%, respectively. Among the 20 CBSAs with the largest estimated MSM populations (Table 4), the New York-Newark-Jersey City CBSA accounted for 8.0% of the national MSM population (n = 167 370). The Los Angeles-Long Beach-Anaheim CBSA followed with 103 044 MSM (4.9%) and then the Miami-Fort Lauderdale-West Palm Beach CBSA with 71 712 MSM (3.4%). These top 20 CBSAs accounted for >50% of the total MSM population nationwide. The highest proportions of MSM relative to adult male population within CBSAs were observed in San Francisco-Oakland-Hayward (3.0%) and Miami-Fort Lauderdale-West Palm Beach (3.1%).

**Table 3.** Estimated MSM Populations in 50 US Counties, Ranked by Size of MSM Population Using Prevalence Estimates of Men Who had Sex With Men in the Past 12 Months

Rank	Name	State	Adult Males (N)	MSM (N, %)	% of Total MSM
**1**	Los Angeles County	CA	3 855 252	82 513 (2.1)	3.9
**2**	Cook County	IL	1 994 078	39 580 (2.0)	1.9
**3**	Broward County	FL	735 660	34 321 (4.7)	1.6
**4**	Harris County	TX	1 713 407	30 795 (1.8)	1.5
**5**	Maricopa County	AZ	1 647 258	30 110 (1.8)	1.4
**6**	San Diego County	CA	1 289 231	26 458 (2.1)	1.3
**7**	New York County	NY	676 766	24 739 (3.7)	1.2
**8**	Riverside County	CA	895 184	23 433 (2.6)	1.1
**9**	Miami-Dade County	FL	1 031 792	22 724 (2.2)	1.1
**10**	King County	WA	902 755	21 908 (2.4)	1.0
**11**	Dallas County	TX	963 409	21 230 (2.2)	1.0
**12**	Kings County	NY	991 857	20 730 (2.1)	1.0
**13**	Orange County	CA	1 221 513	20 530 (1.7)	1.0
**14**	San Francisco County	CA	388 550	18 033 (4.6)	0.9
**15**	Clark County	NV	862 894	17 809 (2.1)	0.8
**16**	Queens County	NY	930 381	17 633 (1.9)	0.8
**17**	Middlesex County	MA	634 784	17 412 (2.7)	0.8
**18**	San Bernardino County	CA	796 861	16 759 (2.1)	0.8
**19**	Palm Beach County	FL	571 388	14 666 (2.6)	0.7
**20**	Suffolk County	NY	587 810	14 203 (2.4)	0.7
**21**	Alameda County	CA	648 469	13 814 (2.1)	0.7
**22**	Santa Clara County	CA	766 310	13 363 (1.7)	0.6
**23**	Bexar County	TX	729 955	12 857 (1.8)	0.6
**24**	Orange County	FL	536 287	12 610 (2.4)	0.6
**25**	Philadelphia County	PA	594 462	11 970 (2.0)	0.6
**26**	Tarrant County	TX	758 363	11 923 (1.6)	0.6
**27**	Travis County	TX	509 707	11 878 (2.3)	0.6
**28**	Contra Costa County	CA	433 575	11 834 (2.7)	0.6
**29**	DeKalb County	GA	275 083	11 302 (4.1)	0.5
**30**	Honolulu County	HI	403 400	11 257 (2.8)	0.5
**31**	Sacramento County	CA	586 805	10 960 (1.9)	0.5
**32**	Pima County	AZ	405 970	10 822 (2.7)	0.5
**33**	Hillsborough County	FL	545 885	10 668 (2.0)	0.5
**34**	Hennepin County	MN	493 389	10 548 (2.1)	0.5
**35**	Fulton County	GA	404 922	10 365 (2.6)	0.5
**36**	Oakland County	MI	489 549	10 228 (2.1)	0.5
**37**	Fairfax County	VA	428 842	10 097 (2.4)	0.5
**38**	Franklin County	OH	491 243	9893 (2.0)	0.5
**39**	Nassau County	NY	523 678	9799 (1.9)	0.5
**40**	District of Columbia	DC	264 627	9709 (3.7)	0.5
**41**	Montgomery County	MD	384 527	9439 (2.5)	0.4
**42**	Wayne County	MI	657 017	9291 (1.4)	0.4
**43**	San Mateo County	CA	298 300	8904 (3.0)	0.4
**44**	Multnomah County	OR	326 508	8761 (2.7)	0.4
**45**	Salt Lake County	UT	436 817	8698 (2.0)	0.4
**46**	Suffolk County	MA	314 635	8688 (2.8)	0.4
**47**	Pierce County	WA	349 015	8552 (2.5)	0.4
**48**	Prince George's County	MD	352 457	8438 (2.4)	0.4
**49**	Allegheny County	PA	488 159	8405 (1.7)	0.4
**50**	Pinellas County	FL	384 276	8382 (2.2)	0.4

Abbreviation: MSM, men who have sex with men.

**Table 4.** The 20 CBSAs With the Largest Estimated MSM Populations, Representing One Half of the US MSM Population and Ranked According to Size of MSM Population, Using Prevalence Estimates of Men Who had Sex With Men in the Past 12 Months

Rank	CBSA	Adult Males (N)	MSM (n, %)	% of Total MSM
1	New York-Newark-Jersey City	7 809 260	167 370 (2.1)	8.0
2	Los Angeles-Long Beach-Anaheim	5 076 765	103 044 (2.0)	4.9
3	Miami-Fort Lauderdale-West Palm Beach	2 338 840	71 712 (3.1)	3.4
4	Chicago-Naperville-Elgin	3 607 920	68 748 (1.9)	3.3
5	Washington-Arlington-Alexandria	2 347 283	56 217 (2.4)	2.7
6	San Francisco-Oakland-Hayward	1 870 337	55 661 (3.0)	2.7
7	Dallas-Fort Worth-Arlington	2 795 943	53 018 (1.9)	2.5
8	Atlanta-Sandy Springs-Roswell	2 203 237	52 805 (2.4)	2.5
9	Philadelphia-Camden-Wilmington	2 338 704	48 711 (2.1)	2.3
10	Boston-Cambridge-Newton	1 900 430	48 188 (2.5)	2.3
11	Houston-The Woodlands-Sugar Land	2 569 702	46 591 (1.8)	2.2
12	Riverside-San Bernardino-Ontario	1 692 045	40 191 (2.4)	1.9
13	Seattle-Tacoma-Bellevue	1 568 855	37 722 (2.4)	1.8
14	Phoenix-Mesa-Scottsdale	1 814 491	33 507 (1.8)	1.6
15	Denver-Aurora-Lakewood	1 147 020	28 669 (2.5)	1.4
16	Detroit-Warren-Dearborn	1 658 761	27 955 (1.7)	1.3
17	San Diego-Carlsbad	1 289 231	26 458 (2.1)	1.3
18	Minneapolis-St. Paul-Bloomington	1 389 513	26 025 (1.9)	1.2
19	Tampa-St. Petersburg-Clearwater	1 219 381	25 706 (2.1)	1.2
20	Orlando-Kissimmee-Sanford	1 002 023	24 603 (2.5)	1.2

Abbreviation: CBSA, core-based statistical areas.

Using prevalence estimates of men who ever had sex with men from NHANES 2015–2020 March [12] (Table 1), we estimated 10 801 491 men who ever had sex with another man in their lifetime, corresponding to an overall prevalence of 8.6% (Supplementary Table 4**).** Among all 50 states and the District of Columbia, California had the largest estimated population of men who ever had sex with another man, with 1 606 697 men, comprising 10.6% of adult males and accounting for 14.9% of the total estimated population of men who ever had sex with another man. Texas (904 294 men; 8.5% of adult males) and Florida (886 415 men; 10.7%) followed, each contributing more than 8% to the national total population of men reported ever having sex with another men. At the county level, Los Angeles County, CA, had the highest estimated population of men who ever had sex with another man, with 450 184 men (11.7% of adult males), accounting for 4.2% of the national total number of men who reported ever having sex with another men (Supplementary Table 5). Other counties with large populations of men who ever had sex with another man included Cook County, IL (215 944; 10.8%), and Harris County, TX (168 014; 9.8%). San Francisco County had the highest county-level prevalence at 25.3% (Supplementary Table 5). Estimates by CBSA showed the New York-Newark-Jersey City area had the largest absolute population of men who ever had sex with another man (808 928; 10.4% of adult males), representing 7.8% of the national population of men who ever had sex with another man. The Los Angeles-Long Beach-Anaheim CBSA followed with 562 195 men(11.1%), and the Chicago-Naperville-Elgin CBSA had 336 260 men (9.3%). The San Francisco-Oakland-Hayward CBSA had the highest prevalence (14.5%) among the top 20 CBSAs (Supplementary Table 6).

The full set of county-, state-, and CBSA-level MSM estimates are provided in Supplementary Tables 7–12.

## DISCUSSION

In this study, we used a previously developed method [7] to update estimates of MSM populations at the county, state, and CBSA levels using the most up-to-date data from 2020 Decennial Census of Population and Housing [11] and NHANES (2015–2020 March) [12]. We examined the MSM percentages by urbanicity and the proportion of SSM households within urbanicity levels and found out that most MSM reside in a relatively limited number of counties and CBSAs. This method offers a straightforward and reliable way to estimate the population sizes of MSM in small areas and can be easily updated with new data as it becomes available [7].

Our findings present 2 analytically distinct behavioral populations: men reporting sex with another man in the past 12 months, which we refer to as MSM, and men with lifetime same-sex experience. These populations capture different dimensions of sexual behavior and are not interchangeable. The past-12-months definition is intended to approximate the population currently at behavioral risk relevant to annual HIV and STI surveillance and program planning. Estimates based on recent sexual activity can help identify individuals at the highest immediate risk, enabling more precise targeting of prevention services such as Pre-exposure prophylaxis (PrEP), testing, and counseling [17]. In contrast, lifetime same-sex experience reflects cumulative behavioral history and represents a broader demographic measure. Lifetime estimates should not be interpreted as representing the population presently at risk of HIV or other STIs, but rather as a measure of historical exposure that may be relevant for descriptive demographic or modeling purposes [18]. Presenting both measures allows readers to select denominators aligned with the epidemiologic question of interest while preserving conceptual clarity about the behavioral meaning of each estimate.

Using sexual behavior reported within the past 12 months, we estimate that approximately 2.1 million MSM reside across all 50 states and the District of Columbia, representing 1.7% of the adult male population. Our methodological approach aligns closely with that of Grey et al.; however, our estimates are lower than those reported by Grey et al [7]. Because our estimates are derived from a different behavioral recall window than Grey et al [7], the absolute population sizes should not be interpreted as directly comparable. Grey et al [7] used a 5-year recall definition of male–male sexual behavior [19], whereas the present analysis uses a past-12-month definition, which intentionally captures a smaller subset of men with more recent sexual activity. Shorter recall periods are expected to yield lower absolute counts, and the approximately 2-fold difference observed for some jurisdictions reflects this methodological distinction rather than a true demographic shift in the MSM population. For surveillance and programmatic use, it is important that denominators align conceptually with the numerator being measured; for example, annual HIV diagnosis rates among men at recent behavioral risk are more appropriately paired with denominators based on recent sexual behavior rather than multiyear or lifetime definitions. Importantly, despite differences in magnitude, the relative geographic distribution of MSM populations remains highly consistent with prior work, suggesting that the spatial patterns of MSM concentration are robust to alternative behavioral definitions.

Our estimate of 1.7% MSM prevalence is lower than the 3.3% reported in Bennett et al.'s recent meta-analysis [6]. This difference is primarily attributable to methodological differences. Although Bennett et al pooled estimates across multiple surveys, some of which reported higher prevalence than NHANES [6], we relied exclusively on restricted NHANES data, which tends to yield lower estimates. Furthermore, our analysis weighted estimates by urbanicity and incorporated the 2015–2016 NHANES cycle, during which reported MSM prevalence was lower than in subsequent cycles. These differences in data sources, time frames, and geographic adjustments likely explain the lower magnitude of our estimate, although the geographic distribution patterns across states and regions remain consistent with prior studies [7].

In contrast, when using a broader definition that includes men who have ever had sex with another man, our estimate rises substantially to 10.8 million men: 8.6% of adult men. Our estimates based on broader lifetime behavior definition are consistent with, and slightly above, earlier national estimates [6], which found that 6.2% of adult men had engaged in same-sex behavior at some point in their lives. The slightly higher prevalence in our data likely reflects updated population denominators, urban migration patterns, and refinements in geographic distribution modeling. It is also possible that patterns of same-sex experience shifted between the earlier survey periods and the time frame of our data, which reflects self-reported behaviors through 2020 [19].

At the county level, our estimates of men who ever had sex with another man support the previously documented geographic concentration of MSM populations in urban centers. For example, San Francisco County, CA, was estimated to have 98 389 men who reported ever having sex with another men, representing 25.3% of its adult male population. This figure is consistent with prior estimates, reported by Grey et al (2009–2013) [7] and Raymond et al (2010) [20], suggesting a persistent demographic pattern over time. Counties such as Broward County, FL, and New York County, NY, continue to rank among the highest in MSM prevalence, reinforcing the durability of urban clustering in the spatial distribution of MSM communities.

It should also be considered that our approach relies on same-sex male partnered households as a proxy for the geographic distribution of MSM, which may not fully capture men who are single, not cohabiting, or unwilling to report same-sex partnerships in Census data. This proxy likely underestimates MSM populations in areas where stigma or privacy concerns suppress reporting and may overrepresent regions where partnered households are more visible, particularly in urban centers. Accordingly, the modeled distribution should be interpreted as an approximation of relative concentration rather than a complete census of MSM residence.

Regional estimates of recent versus lifetime same-sex experience should be interpreted cautiously because NHANES regional subsamples may be relatively small and therefore subject to sampling variability. The discrepancy observed in the Midwest may reflect a combination of sampling fluctuation and underlying migration or partnership patterns, including the possibility that sexually active MSM disproportionately relocate to large metropolitan areas outside the region. These factors highlight that regional behavioral estimates are approximate indicators rather than precise measures of short-term activity.

Challenges in estimating MSM population denominators are not unique to the United States and have been documented internationally. Studies from the United Kingdom and Switzerland describe similar methodological tensions between behavioral surveys, household-based proxies, and the persistent “denominator problem” in estimating sexual minority populations [21, 22]. Differences in survey design, cultural reporting norms, and administrative data systems limit direct numerical comparison across countries; however, the conceptual framework underlying our approach, such as combining behavioral prevalence with geographic weighting, parallels strategies used in other industrialized settings. These parallels suggest that the methodological issues addressed here reflect broader international challenges rather than U.S.-specific phenomena.

In the current analysis, we made some modifications to our previous method that may result in possible limitations. We collapsed the “small metropolitan, micropolitan, and noncore” urbanicity levels to prevent risks related to confidentiality disclosure. Collapsing micropolitan and noncore counties into a single rural category may obscure heterogeneity across rural communities and reduce precision in less populated areas. This aggregation was required to satisfy confidentiality and minimum cell size constraints in the restricted NHANES data and represents a tradeoff between geographic granularity and data protection. Second, NHANES sexual behavior questions are restricted to adults younger than age 60 years, whereas Census denominators include all adults aged 18 years and older. Because no national dataset provides comparable behavioral estimates for older age groups with sufficient geographic detail, we applied NHANES-derived prevalence estimates to the full adult male population. If same-sex behavior is less frequently reported among men aged 60 years and older, this approach may modestly overestimate MSM population size; however, the magnitude of this bias is likely small relative to total population estimates. Behavioral prevalence estimates of male–male sexual contact may vary across national surveys because of differences in sampling design, questionnaire context, and measurement approaches. We relied on restricted NHANES data because they provide the geographic identifiers required for our county-level MSM estimates calculations. Future methodological work could evaluate how alternative survey inputs influence downstream small-area population estimates. Also, uncertainty from NHANES prevalence estimates was not propagated through to county- and state-level estimates. County- and state-level MSM counts should be interpreted as modeled point estimates rather than exact population counts, and uncertainty is expected to be greater in sparsely populated areas. Future work using simulation-based approaches could formally quantify uncertainty around small-area MSM estimates.

## CONCLUSIONS

We used the most recent data from the Decennial Census and NHANES to update estimates of the MSM population. These estimates at the county, state, and CBSA levels are essential for evaluating the effects of HIV and STIs among MSM in smaller geographic areas and for formulating prevention and treatment strategies. Our approach allows for the regular updating of these MSM estimates when new decennial census and NHANES data are released, providing counties and larger regions with up-to-date population figures and potentially revised incidence and prevalence rates. MSM population estimates at the county, state, and CBSA levels can improve the utility of epidemiologic measures of HIV and STI epidemics, guide the allocation of resources, inform the planning of interventions, and optimize delivery of services to reduce HIV and STIs among US MSM.

## Supplementary Material

ofag148_Supplementary_Data

## Supplementary Data

Supplementary materials are available at *Open Forum Infectious Diseases* online. Consisting of data provided by the authors to benefit the reader, the posted materials are not copyedited and are the sole responsibility of the authors, so questions or comments should be addressed to the corresponding author.
