# Supplementary material for: County- and State-level Estimates of Population Sizes of Men Who Have Sex With Men Across the United States
Source: Open Forum Infect Dis. 2026 Apr 14;13(4):ofag148. doi: 10.1093/ofid/ofag148 (PMC13077674; doi:10.1093/ofid/ofag148)
Supplement: ofag148_Supplementary_Data [file ofag148_supplementary_data.zip › Supplementary_Tables 1_6.docx]

**Supplementary Table 1.** Variable names, descriptions and their locations in publicly available data files of 2020 Decennial Census of Population and Housing

| **Variable name** | **Variable description** | **Data file name** | **Data file description** | **Year** |
| --- | --- | --- | --- | --- |
| PCT15_010N | Male-male unmarried partner households | DECENNIALDHC2020.PCT-15 | Population and Housing | 2020 |
| PCT15_005N | Male-male married couple households | DECENNIALDHC2020.PCT-15 | Population and Housing | 2020 |
| PCT15_001N | Total number of households | DECENNIALDHC2020.PCT-15 | Population and Housing | 2020 |
| DP1_0045C | Male population, 18 years and over | DECENNIALDP2020.DP1 | Profile of General Population | 2020 |

**Supplementary Table 2.** Variable names, descriptions and their locations in publicly available National Health and Nutrition Examination Survey (NHANES) data (2015-2020 March)

| **Variable name** | **Variable description** | **Data file name** | **Data file description** | **Begin year** | **End year** |
| --- | --- | --- | --- | --- | --- |
| SEQN | Respondent sequence number. | DEMO_I | Demographic Variables and Sample Weights | 2015 | 2016 |
| SDDSRVYR | Data release cycle | DEMO_I | Demographic Variables and Sample Weights | 2015 | 2016 |
| WTINT2YR | Full sample 2-year interview weight | DEMO_I | Demographic Variables and Sample Weights | 2015 | 2016 |
| WTMEC2YR | Full sample 2-year MEC exam weight | DEMO_I | Demographic Variables and Sample Weights | 2015 | 2016 |
| RIDAGEYR | Age in years of the participant at the time of screening. Individuals 80 and over are top coded at 80 years of age. | DEMO_I | Demographic Variables and Sample Weights | 2015 | 2016 |
| RIAGENDR | Gender of the participant. | DEMO_I | Demographic Variables and Sample Weights | 2015 | 2016 |
| SEQN | Respondent sequence number. | P_DEMO | Demographic Variables and Sample Weights | 2017 | March 2020 |
| SDDSRVYR | Data release cycle | P_DEMO | Demographic Variables and Sample Weights | 2017 | March 2020 |
| WTINTPRP | Full sample interview weight | P_DEMO | Demographic Variables and Sample Weights | 2017 | March 2020 |
| WTMECPRP | Full sample MEC exam weight | P_DEMO | Demographic Variables and Sample Weights | 2017 | March 2020 |
| RIDAGEYR | Age in years of the participant at the time of screening. Individuals 80 and over are top coded at 80 years of age. | P_DEMO | Demographic Variables and Sample Weights | 2017 | March 2020 |
| RIAGENDR | Gender of the participant. | P_DEMO | Demographic Variables and Sample Weights | 2017 | March 2020 |

**Supplementary Table 3.** Variable names, descriptions and their locations in restricted-use adult sexual behavior questionnaire in National Health and Nutrition Examination Survey (NHANES) data (2015-2020 March)

| **Variable name** | **Variable description** | **Data file name** | **Data file description** | **Begin year** | **End year** |
| --- | --- | --- | --- | --- | --- |
| SXQ809 | Have you ever had any kind of sex with a man, including oral or anal? | P_SXQ_R | Sexual Behavior - Adult | 2015 | 2016 |
| SXQ550 | In the past 12 months, with how many men have you had anal or oral sex? | P_SXQ_R | Sexual Behavior - Adult | 2015 | 2016 |
| SXQ809 | Have you ever had any kind of sex with a man, including oral or anal? | P_SXQ_R | Sexual Behavior - Adult | 2017 | 2018-March 2020 |
| SXQ550 | In the past 12 months, with how many men have you had anal or oral sex? | P_SXQ_R | Sexual Behavior - Adult | 2017 | 2018-March 2020 |

**Supplementary Table 4:** Estimated MSM populations in 50 states and the District of Columbia, ranked by size of MSM population using prevalence estimates of men of men who ever had sex with men*

| **Rank** | **State** | **Adult males (n)** | **MSM (n, %)** | **% of total MSM** |
| --- | --- | --- | --- | --- |
| 1 | California | 15,092,993 | 1,606,697 (10.6) | 14.9 |
| 2 | Texas | 10,676,141 | 904,294 (8.5) | 8.4 |
| 3 | Florida | 8,318,714 | 886,415 (10.7) | 8.2 |
| 4 | New York | 7,666,027 | 769,674 (10.0) | 7.1 |
| 5 | Illinois | 4,845,934 | 407,981 (8.4) | 3.8 |
| 6 | Pennsylvania | 5,005,473 | 391,778 (7.8) | 3.6 |
| 7 | Georgia | 3,918,344 | 338,085 (8.6) | 3.1 |
| 8 | Ohio | 4,456,327 | 326,150 (7.3) | 3.0 |
| 9 | New Jersey | 3,492,639 | 306,690 (8.8) | 2.8 |
| 10 | North Carolina | 3,901,719 | 297,142 (7.6) | 2.8 |
| 11 | Washington | 2,983,509 | 291,168 (9.8) | 2.7 |
| 12 | Massachusetts | 2,702,673 | 279,682 (10.3) | 2.6 |
| 13 | Michigan | 3,864,050 | 272,245 (7.0) | 2.5 |
| 14 | Virginia | 3,255,740 | 263,997 (8.1) | 2.4 |
| 15 | Arizona | 2,715,833 | 247,166 (9.1) | 2.3 |
| 16 | Colorado | 2,250,459 | 204,683 (9.1) | 1.9 |
| 17 | Maryland | 2,279,740 | 203,809 (8.9) | 1.9 |
| 18 | Tennessee | 2,587,094 | 191,332 (7.4) | 1.8 |
| 19 | Indiana | 2,529,026 | 182,025 (7.2) | 1.7 |
| 20 | Missouri | 2,317,242 | 169,955 (7.3) | 1.6 |
| 21 | Minnesota | 2,161,621 | 162,892 (7.5) | 1.5 |
| 22 | Oregon | 1,652,902 | 150,844 (9.1) | 1.4 |
| 23 | Wisconsin | 2,275,140 | 150,798 (6.6) | 1.4 |
| 24 | South Carolina | 1,910,038 | 132,324 (6.9) | 1.2 |
| 25 | Nevada | 1,198,598 | 123,588 (10.3) | 1.1 |
| 26 | Louisiana | 1,707,087 | 122,933 (7.2) | 1.1 |
| 27 | Kentucky | 1,691,374 | 118,230 (7.0) | 1.1 |
| 28 | Connecticut | 1,373,574 | 117,496 (8.6) | 1.1 |
| 29 | Alabama | 1,861,340 | 115,399 (6.2) | 1.1 |
| 30 | Oklahoma | 1,475,525 | 105,188 (7.1) | 1.0 |
| 31 | Utah | 1,156,059 | 91,213 (7.9) | 0.8 |
| 32 | Iowa | 1,207,151 | 73,685 (6.1) | 0.7 |
| 33 | Kansas | 1,099,110 | 73,497 (6.7) | 0.7 |
| 34 | Arkansas | 1,118,227 | 71,516 (6.4) | 0.7 |
| 35 | New Mexico | 799,990 | 68,820 (8.6) | 0.6 |
| 36 | Hawaii | 573,981 | 60,772 (10.6) | 0.6 |
| 37 | Mississippi | 1,081,825 | 60,161 (5.6) | 0.6 |
| 38 | District of Columbia | 264,627 | 52,969 (20.0) | 0.5 |
| 39 | Maine | 538,251 | 45,410 (8.4) | 0.4 |
| 40 | New Hampshire | 550,142 | 45,335 (8.2) | 0.4 |
| 41 | Nebraska | 723,943 | 45,012 (6.2) | 0.4 |
| 42 | West Virginia | 703,779 | 44,269 (6.3) | 0.4 |
| 43 | Rhode Island | 424,360 | 41,763 (9.8) | 0.4 |
| 44 | Idaho | 681,530 | 40,365 (5.9) | 0.4 |
| 45 | Delaware | 371,822 | 39,421 (10.6) | 0.4 |
| 46 | Montana | 421,606 | 22,069 (5.2) | 0.2 |
| 47 | Vermont | 256,089 | 21,063 (8.2) | 0.2 |
| 48 | Alaska | 283,116 | 19,292 (6.8) | 0.2 |
| 49 | South Dakota | 332,921 | 17,091 (5.1) | 0.2 |
| 50 | North Dakota | 303,212 | 15,433 (5.1) | 0.1 |
| 51 | Wyoming | 222,567 | 11,675 (5.2) | 0.1 |
|  | Total | 125,281,184 | 10,801,491 (8.6) |  |

* State-level estimates were created by applying National Health and Nutrition Examination Survey (NHANES)urbanicity-level estimates of men who have sex with men to 2020 Decennial Census county-level estimates of same-sex male households.

**Supplementary Table 5:** Estimated MSM populations in 50 US counties, ranked by size of MSM population prevalence estimates of men who ever had sex with men

| **Rank** | **County** | **State** | **Adult males (n)** | **MSM (n, %)** | **% of total MSM** |
| --- | --- | --- | --- | --- | --- |
| 1 | Los Angeles County | CA | 3,855,252 | 450,184 (11.7) | 4.2 |
| 2 | Cook County | IL | 1,994,078 | 215,944 (10.8) | 2.0 |
| 3 | Harris County | TX | 1,713,407 | 168,014 (9.8) | 1.6 |
| 4 | Maricopa County | AZ | 1,647,258 | 164,278 (10.0) | 1.5 |
| 5 | San Diego County | CA | 1,289,231 | 144,352 (11.2) | 1.3 |
| 6 | Broward County | FL | 735,660 | 141,575 (19.2) | 1.3 |
| 7 | New York County | NY | 676,766 | 134,976 (19.9) | 1.2 |
| 8 | Riverside County | CA | 895,184 | 127,846 (14.3) | 1.2 |
| 9 | Miami-Dade County | FL | 1,031,792 | 123,980 (12.0) | 1.1 |
| 10 | King County | WA | 902,755 | 119,529 (13.2) | 1.1 |
| 11 | Dallas County | TX | 963,409 | 115,829 (12.0) | 1.1 |
| 12 | Kings County | NY | 991,857 | 113,101 (11.4) | 1.0 |
| 13 | Orange County | CA | 1,221,513 | 112,011 (9.2) | 1.0 |
| 14 | San Francisco County | CA | 388,550 | 98,389 (25.3) | 0.9 |
| 15 | Clark County | NV | 862,894 | 97,165 (11.3) | 0.9 |
| 16 | Queens County | NY | 930,381 | 96,203 (10.3) | 0.9 |
| 17 | Alameda County | CA | 648,469 | 75,368 (11.6) | 0.7 |
| 18 | Santa Clara County | CA | 766,310 | 72,910 (9.5) | 0.7 |
| 19 | Middlesex County | MA | 634,784 | 71,826 (11.3) | 0.7 |
| 20 | Bexar County | TX | 729,955 | 70,144 (9.6) | 0.6 |
| 21 | San Bernardino County | CA | 796,861 | 69,130 (8.7) | 0.6 |
| 22 | Orange County | FL | 536,287 | 68,799 (12.8) | 0.6 |
| 23 | Philadelphia County | PA | 594,462 | 65,306 (11.0) | 0.6 |
| 24 | Tarrant County | TX | 758,363 | 65,049 (8.6) | 0.6 |
| 25 | Travis County | TX | 509,707 | 64,803 (12.7) | 0.6 |
| 26 | Palm Beach County | FL | 571,388 | 60,499 (10.6) | 0.6 |
| 27 | Sacramento County | CA | 586,805 | 59,797 (10.2) | 0.6 |
| 28 | Suffolk County | NY | 587,810 | 58,588 (10.0) | 0.5 |
| 29 | Hillsborough County | FL | 545,885 | 58,203 (10.7) | 0.5 |
| 30 | Hennepin County | MN | 493,389 | 57,546 (11.7) | 0.5 |
| 31 | Fulton County | GA | 404,922 | 56,550 (14.0) | 0.5 |
| 32 | Franklin County | OH | 491,243 | 53,977 (11.0) | 0.5 |
| 33 | District of Columbia | DC | 264,627 | 52,969 (20.0) | 0.5 |
| 34 | Wayne County | MI | 657,017 | 50,689 (7.7) | 0.5 |
| 35 | Contra Costa County | CA | 433,575 | 48,817 (11.3) | 0.5 |
| 36 | Multnomah County | OR | 326,508 | 47,801 (14.6) | 0.4 |
| 37 | Salt Lake County | UT | 436,817 | 47,456 (10.9) | 0.4 |
| 38 | Suffolk County | MA | 314,635 | 47,400 (15.1) | 0.4 |
| 39 | DeKalb County | GA | 275,083 | 46,621 (16.9) | 0.4 |
| 40 | Allegheny County | PA | 488,159 | 45,856 (9.4) | 0.4 |
| 41 | Pinellas County | FL | 384,276 | 45,732 (11.9) | 0.4 |
| 42 | Denver County | CO | 290,114 | 42,964 (14.8) | 0.4 |
| 43 | Bronx County | NY | 511,489 | 42,651 (8.3) | 0.4 |
| 44 | Oakland County | MI | 489,549 | 42,191 (8.6) | 0.4 |
| 45 | Fairfax County | VA | 428,842 | 41,650 (9.7) | 0.4 |
| 46 | Honolulu County | HI | 403,400 | 41,452 (10.3) | 0.4 |
| 47 | Mecklenburg County | NC | 407,212 | 41,039 (10.1) | 0.4 |
| 48 | Cuyahoga County | OH | 474,203 | 40,510 (8.5) | 0.4 |
| 49 | Nassau County | NY | 523,678 | 40,419 (7.7) | 0.4 |
| 50 | Pima County | AZ | 405,970 | 39,849 (9.8) | 0.4 |

**Supplementary Table 6:** The 20 CBSAs with the largest estimated MSM populations, ranked according to size of MSM population, using prevalence estimates of men who ever had sex with men

| **Rank** | **CBSA*** | **Adult males (N)** | **MSM (n, %)** | **% of total MSM** |
| --- | --- | --- | --- | --- |
| 1 | New York-Newark-Jersey City | 7,809,260 | 808,928 (10.4) | 7.8 |
| 2 | Los Angeles-Long Beach-Anaheim | 5,076,765 | 562,195 (11.1) | 5.4 |
| 3 | Chicago-Naperville-Elgin | 3,607,920 | 336,260 (9.3) | 3.2 |
| 4 | Miami-Fort Lauderdale-West Palm Beach | 2,338,840 | 326,054 (13.9) | 3.1 |
| 5 | San Francisco-Oakland-Hayward | 1,870,337 | 271,988 (14.5) | 2.6 |
| 6 | Dallas-Fort Worth-Arlington | 2,795,943 | 270,743 (9.7) | 2.6 |
| 7 | Washington-Arlington-Alexandria | 2,347,283 | 249,894 (10.6) | 2.4 |
| 8 | Houston-The Woodlands-Sugar Land | 2,569,702 | 233,171 (9.1) | 2.2 |
| 9 | Atlanta-Sandy Springs-Roswell | 2,203,237 | 231,616 (10.5) | 2.2 |
| 10 | Philadelphia-Camden-Wilmington | 2,338,704 | 216,862 (9.3) | 2.1 |
| 11 | Boston-Cambridge-Newton | 1,900,430 | 210,336 (11.1) | 2.0 |
| 12 | Riverside-San Bernardino-Ontario | 1,692,045 | 196,976 (11.6) | 1.9 |
| 13 | Seattle-Tacoma-Bellevue | 1,568,855 | 184,761 (11.8) | 1.8 |
| 14 | Phoenix-Mesa-Scottsdale | 1,814,491 | 178,289 (9.8) | 1.7 |
| 15 | San Diego-Carlsbad | 1,289,231 | 144,352 (11.2) | 1.4 |
| 16 | Tampa-St. Petersburg-Clearwater | 1,219,381 | 131,392 (10.8) | 1.3 |
| 17 | Denver-Aurora-Lakewood | 1,147,020 | 128,742 (11.2) | 1.2 |
| 18 | Detroit-Warren-Dearborn | 1,658,761 | 127,679 (7.7) | 1.2 |
| 19 | Minneapolis-St. Paul-Bloomington | 1,389,513 | 126,201 (9.1) | 1.2 |
| 20 | Orlando-Kissimmee-Sanford | 1,002,023 | 118,269 (11.8) | 1.1 |

*CBSA: Core-Based Statistical Areas
